# Experimental hemodialysis in diet-induced ketosis and the potential use of dialysis as an adjuvant cancer treatment

**DOI:** 10.1038/s41598-023-46715-7

**Published:** 2023-11-09

**Authors:** Carl M. Öberg, Jan Sternby, Anders Nilsson, Markus Storr, Ralf Flieg, Kai Harenski, Viktoria Roos, Linda Källquist, Sture Hobro

**Affiliations:** 1 Department of Clinical Sciences Lund, Skåne University Hospital, Njurmottagningen SUS Lund, Barngatan 2a, 221 85 Lund, Sweden; 2 Baxter International Inc., Magistratsvägen 10, 22643 Lund, Sweden; 3 Baxter International Inc., 72379 Hechingen, Germany; 4 Baxter Deutschland GmbH., 85 716 Unterschleissheim, Germany

**Keywords:** Haemodialysis, Cancer therapy, Physiology, Kidney

## Abstract

Numerous in vivo studies on the ketogenic diet, a diet that can induce metabolic conditions resembling those following extended starvation, demonstrate strong outcomes on cancer survival, particularly when combined with chemo-, radio- or immunological treatments. However, the therapeutic application of ketogenic diets requires strict dietary adherence from well-informed and motivated patients, and it has recently been proposed that hemodialysis might be utilized to boost ketosis and further destabilize the environment for cancer cells. Yet, plasma ketones may be lost in the dialysate—lowering blood ketone levels. Here we performed a single 180-min experimental hemodialysis (HD) session in six anesthetized Sprague–Dawley rats given ketogenic diet for five days. Median blood ketone levels pre-dialysis were 3.5 mmol/L (IQR 2.2 to 5.6) and 3.8 mmol/L (IQR 2.2 to 5.1) after 180 min HD, *p* = 0.54 (95% CI − 0.6 to 1.2). Plasma glucose levels were reduced by 36% (− 4.5 mmol/L), *p* < 0.05 (95% CI − 6.7 to − 2.5). Standard base excess was increased from − 3.5 mmol/L (IQR − 4 to − 2) to 0.5 mmol/L (IQR − 1 to 3), *p* < 0.01 (95% CI 2.0 to 5.0). A theoretical model was applied confirming that intra-dialytic glucose levels decrease, and ketone levels slightly increase since hepatic ketone production far exceeds dialytic removal. Our experimental data and *in-silico* modeling indicate that elevated blood ketone levels during ketosis are maintained during hemodialysis despite dialytic removal.

## Introduction

Cancer currently poses the largest medical, social and economic disease burden worldwide^1^. Therapy options for many common malignancies remain limited, and treatment resistance further reduces efficacy of existing agents. Hemodialysis could potentially be used as an adjunct therapy in addition to traditional cancer therapies to improve survival for malignant cancers with poor prognosis^2^.

For most cancers, surgery, radiation, and immune- or chemotherapy remain standard of care. In addition, a ketogenic diet (a low-carbohydrate, high-fat diet with sufficient protein) has been reported to improve efficacy of existing anti-cancer therapies^3^. Recently, we suggested that dialysis might be used to change the body's metabolism to a pronounced ketogenic state that is similar to the metabolic conditions attained after prolonged starvation, preventing the issues that vulnerable cancer patients might experience with prolonged starvation or a specialized diet^2^. Indeed, lower insulin and glucose levels can easily be accomplished with a dialysate free of glucose and a proper diet before and during dialysis^4^. Wathen et al. found that ketones increased significantly during glucose-free dialysis and concluded that the fasting dialysis patient undergoing glucose-free dialysis resembles an otherwise normal individual who has undergone fasting of about 48 to 72 h^5^.

Modern high-flux hemodialyzers efficiently remove small molecular substances such as glucose and amino acids, and also larger molecules^6^ similar to the size of small peptide hormones such as insulin, and to a certain degree even plasma albumin^7^. If the patient is treated with ketogenic diet, hemodialysis could thus be used to further enhance the hypoinsulinemia and ketogenically compensated glucose availability associated with ketosis. However, dialysis would also readily remove ketone bodies, suppressing ketosis. Here we performed experimental hemodialysis in Sprague–Dawley rats pre-treated with ketogenic diet for five days. We also applied a theoretical model with concentrations adapted to male Wistar rats to mimic a similar treatment in humans, and to elucidate the mechanisms involved. Flows and organ volumes are relevant for a human dialysis treatment setting.

## Methods

### Animals

Experiments were performed in six male Sprague–Dawley rats having an average body weight of 316 g (305–318 g) given water and ketogenic food (Kliba-Nafag 2201 Ketogenic diet XL75:XP10) five days prior to experiments. Before commencing ketogenic diet, rats were fed standard food (Special Diets Services RM1(P) IRR.25 801157). The animals were treated according to the guidelines of the National Institutes of Health for Care and Use of Laboratory animals. The Ethics Committee for Animal Research at Lund University approved of the experiments (Dnr 5.8.18-08386/2022). Results are reported in compliance with the ARRIVE guidelines. No specific exclusion or inclusion criteria were used. The rat was carefully placed into a covered glass container to which a continuous supply of 5% isoflurane in air (Isoban, Abbot Stockholm, Sweden) was connected. After fully anesthetized, the rat was gently lifted from the container. Anesthesia was maintained using 1.6–1.8% isoflurane in air delivered in a small mask. Following tracheostomy, the rat was connected to a volume-controlled ventilator (Ugo Basile; Biological Research Apparatus, Comerio, Italy) using a positive end-expiratory pressure of 4 cm H_2_O. Body temperature was kept between 37.1 °C and 37.3 °C by using a feedback-controlled heating pad. End-tidal *p*CO_2_ was maintained between 4.8 and 5.5 kPa (Capstar-100, CWE, Ardmore, Pa). The right femoral artery was cannulated for continuous monitoring of heart rate and mean arterial pressure (MAP); and for obtaining blood samples (95 μL) for measurement of glucose, urea, electrolytes, hemoglobin, and hematocrit (I-STAT, Abbott, Abbott Park, IL), and blood ketones (FreeStyle Precision Neo, Abbott, Abbott Park, IL). The right femoral vein was cannulated and connected to the dialyzer using plastic tubing. The right femoral artery was also cannulated and connected both to a pressure transducer to continuously monitor arterial line pressure, and to a blood pump (Masterflex Ismatec, Cole-Parmer, IL) connected to the dialyzer via plastic tubing. Before connection, the blood circuit was primed with 4% albumin (Albunorm, Octapharma Nordic AB, Sweden) to which heparin 50 IE (Heparin LEO 5000 IE/mL, Leo Pharma AB, Sweden) had been added. The right internal jugular vein was cannulated for infusion of maintenance fluid containing ^51^Cr-EDTA. Hematocrit was determined by centrifugating thin capillary glass tubes. After the experiment, animals were euthanized with an intravenous bolus injection of potassium chloride.

### Experimental protocol

**Figure 1:**
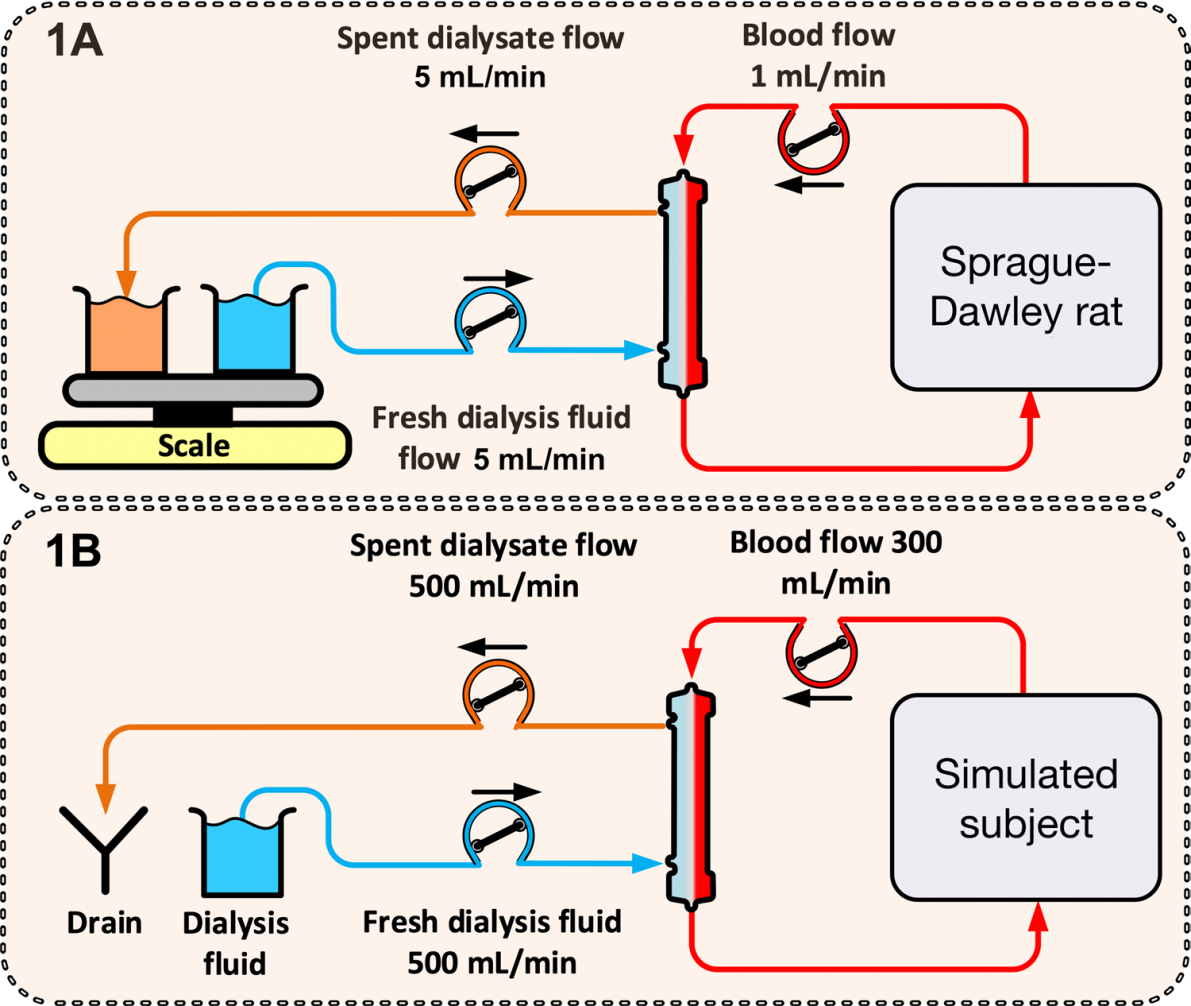
Schematic diagram of the experimental setup (**A**). Hemodialysis was performed in anesthetized ketotic Sprague–Dawley rats using a blood flow rate of 1 mL/min and a dialysate flow rate of 5 mL/min. Schematic diagram of the simulation setup (**B**). Hemodialysis was simulated for a patient using a blood flow rate of 300 mL/min and a dialysate flow rate of 500 mL/min.

The dialysis circuit inlet was connected via a pump to a glass cylinder containing fresh dialysis fluid (Hemosol B0, Baxter Healthcare, IL). The outlet from the dialyzer was also connected to a peristaltic pump which pumped spent dialysate to a glass cylinder. Both glass cylinders were placed on the same scale to ensure that no fluid was removed from the animal (Fig. 1A). A three-hour hemodialysis session was performed using a mini-capillary dialyzer device obtained from Baxter Healthcare (Hechingen, Germany), comprising a high-flux membrane (Polyflux Revaclear ™, HF-Revaclear ™). The membrane had a total surface area of 318 cm^2^ with a total number of 355 fibers, each of which with an inner diameter of 190 μm, and a wall thickness of 35 μm (total intracapillary volume: 1.50 mL). Arterial blood samples were obtained before and 30 min after dialysis, and at 60, 90, 120, and 180 min during HD. Dialysate samples were collected before dialysis and at 10, 20, 40, 60, 90, 120, 150, and 180 min and analyzed on a gamma counter (Wizard 1480, LKP Wallac, Turku, Finland) to determine ^51^Cr-EDTA activity.

### Statistical methods

Data are shown as median (interquartile range) unless otherwise specified. Significant differences were assessed using an asymptotic Friedman omnibus test (coin package) which, if significant, was followed by a Wilcoxon-Nemenyi-McDonald-Thompson post hoc test. Two blood ketone values were above the range of measurement (8.0 mmol/L). Before statistical analysis they were set to 8.0 mmol/L. Since they were the highest values in the dataset, a value of 8 mmol/L ensures that (i) the median (IQR) is unaffected, and (ii) that the datapoints will get the highest and the same rank. P-values below 5% were considered significant. A simple power analysis (pwr.t.test, pwr package) showed that a group size of six animals has 80% power to detect an effect size (Cohen's *d*) of 1.4. For example, for blood ketones, if assuming a SD of 1 mM, such an effect size would correspond to a difference of 1.4 mM. Calculations were performed using R for mac version 4.1.1.

### Simulations

To simulate the effects of dialysis on the systemic blood composition, we used the model developed by Todd^8,9^, in which the evolution of forty-six substances in human blood, liver, fat and muscle tissue was simulated during varying glucose feeding. Even though this is a rodent model we adapted the volumes and flow rates to human conditions. The differential equations for blood concentrations of glucose, insulin, free fatty acids (FFA), ketones, and alanine were modified by introducing a dialysis clearing effect proportional to the concentration difference between blood and dialysis fluid. Although glucose is distributed into erythrocytes^10,11^, the transport is rather slow so that almost only plasma water will be cleared during the blood passage along the dialyzer fibers^12^. Dialyzer plasma water clearance was calculated assuming a plasma water flow rate of 180 mL/min in a blood flow rate of 300 mL/min and a dialysis fluid flow rate of 500 mL/min for a large dialyzer with a mass transfer area coefficient (K_0_A) for urea at 1500 mL/min (see Fig. 1B). The K_0_A value for FFA (*K*_*0*_*A*_*FFA*_) was determined in relation to that of phosphate (*K*_*0*_*A*_*Phos*_ = 0.547 * *K*_*0*_*A*_*urea*_) based on the ratio of molecular weights for phosphate (*MW*_*Phos*_ = 95) and FFA (*MW*_*FFA*_ = 256) according to the equation

$${{K}_{0}A}_{FFA}={{K}_{0}A}_{Phos}*{\left(\frac{{MW}_{Phos}}{{MW}_{FFA}}\right)}^{0.37}$$

All other K_0_A values were determined using the same equation, but relating to K_0_A for creatinine (*K*_*0*_*A*_*crea*_ = 0.638 * *K*_*0*_*A*_*urea*_) and *MW*_*crea*_ = 113. The distribution volumes were assumed to be whole blood for glucose^12^ and plasma water for all other substances. Simulations were performed under the assumption of no intake of nutrients.

## Results

### Baseline parameters

Six Sprague–Dawley rats were given ketogenic diet for five days and gained between (min–max) 10 to 37 g in body weight. Before dialysis, median arterial pH was 7.43 (7.42 to 7.44), actual bicarbonate was 20.4 mmol/L (19.8 to 21.3), standard base excess was − 3.5 mmol/L (− 4.0 to − 2.2), *p*O_2_ was 12.4 kPa (12.2 to 12.7), and *p*CO2 was 4.0 kPa (4.0 to 4.3). Dialysis was performed for three hours after which blood was returned to the animal, followed by a 30 min rest period before termination of the experiment. Treatment and baseline parameters are shown in Tables 1, 2.

**Table 1:** Baseline parameters.

Parameter	Value
Mean arterial pressure, mmHg	69 (62–73)
Heart rate	329 (315–356)
Arterial oxygen saturation (SO_2_) %	98 (97–98)
Plasma sodium, mmol/L	138.5 (138.0–139.8)
Plasma potassium, mmol/L	3.8 (3.8–3.9)
Plasma chloride, mmol/L	103 (103–103)
Plasma ionized calcium, mmol/L	1.28 (1.27–1.34)
Plasma lactate, mmol/L	2.9 (2.1–3.9)

**Table 2:** Treatment parameters.

Parameter	Value
Blood flow (Qb), mL/min	1.0
Dialysate flow (Qd), mL/min	5.0
Dialysate sodium, mM ^a^	140
Dialysate potassium, mM^b^	4
Dialysate bicarbonate, mM^a^	32
Dialysate calcium, mM^a^	1.75
Dialysate lactate, mM^a^	3

^a^Nominal values from the manufacturer (Hemosol B0, Baxter Healthcare, IL).

^b^Approximate value, 20 mmol potassium (Addex-Kalium, Fresenius Kabi, Sweden) was added to 5 L of fresh dialysis fluid.

### Hemodialysis does not significantly reduce blood ketone levels

**Figure 2:**
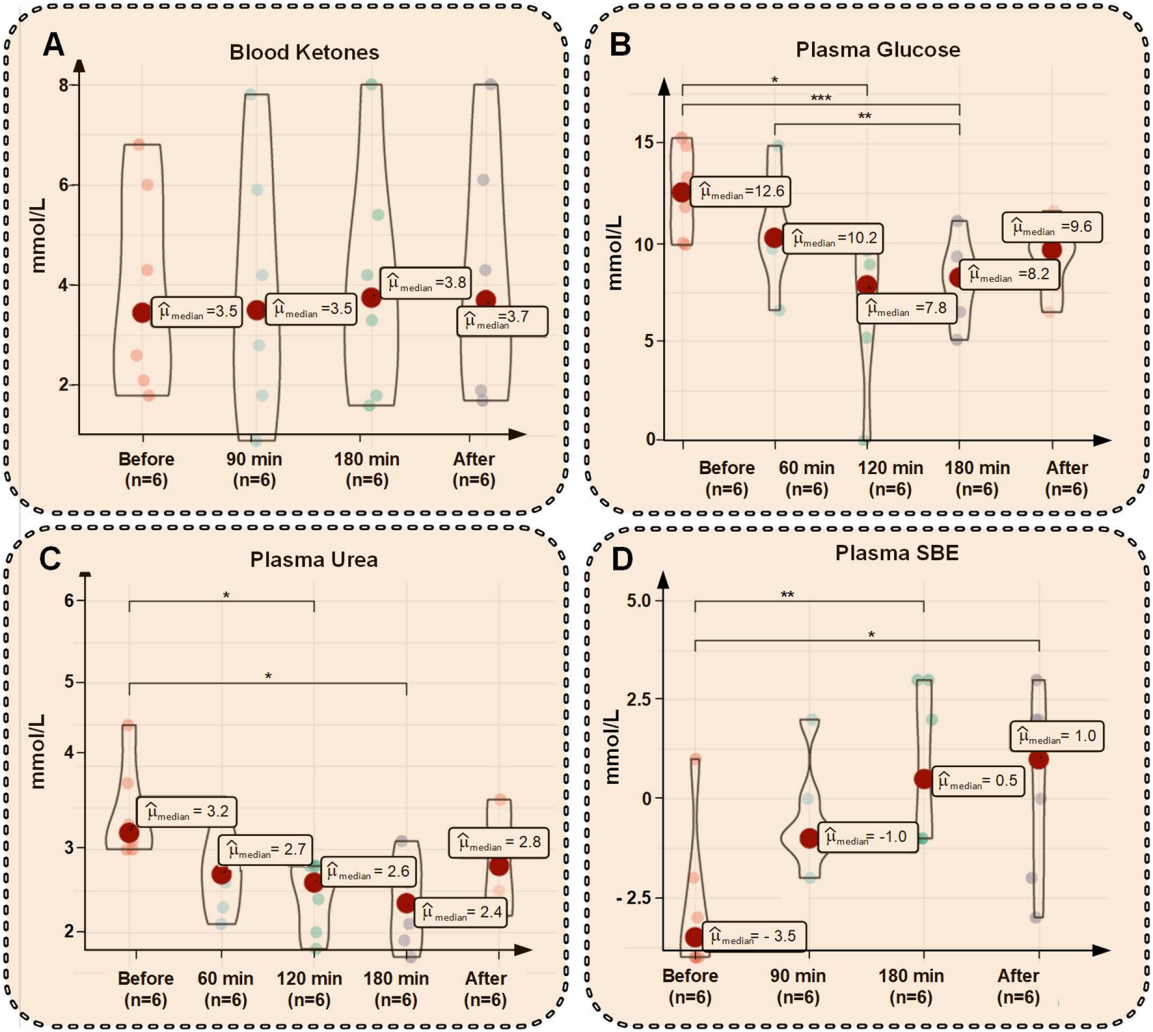
Violin plots of blood ketones (**A**), plasma glucose (**B**), plasma urea (**C**), and standard base excess (SBE) (**D**) immediately before, during, and 30 min after the dialysis session.

Blood ketone levels before and after 180 min dialysis were similar, being 3.5 mmol/L (2.2–5.6) and 3.8 mmol/L (2.2–5.1), respectively (P = 0.53), with no significant differences in ketone concentrations during dialysis (Fig. 2A) (Table 3). Urea reduction ratio (URR) was 38% (25–42) and glucose reduction ratio was 36% (29–43), with concentrations decreasing during the treatment (Fig. 2B,C) (Table 4). The blood to dialysate clearance of ^51^Cr-EDTA was stable during the entire dialysis session being 1.0 mL/min (0.7–1.1). Single-pool Kt/V urea was 0.57 (0.52–0.63), calculated as follows Kt/V = − log(1− URR-0.024). Assuming a total body water of 70% of body weight, this implies a urea clearance of 0.67 mL/min (0.62 to 0.80).

**Table 3:** Effects of dialysis on blood ketones, lactate, and actual HCO_3_.

Group	Ketones (mmol L^−1^)	Lactate (mmol L^−1^)	Actual Bicarbonate (mmol L^−1^)
Before dialysis	3.5 (2.2–5.6)	2.9 (2.0–3.9)	20.4 (19.8–21.3)
90 min	3.5 (2.0–5.5)	1.9 (1.3–2.8)	22.8 (22.4–24.2)
180 min	3.8 (2.2–5.1)	2.0 (1.9–2.2)	23.5 (21.4–24.9)
30 min after dialysis	3.7 (2.2–5.6)	1.6 (1.5–1.8)	23.5 (22.1–25.0)
Asymptotic Friedman test			
P-value	0.49	0.39	0.02
95% CI for dialysis effect	[− 0.2 to 0.4]	[− 0.3 to 0.5]	[0.3 to 0.6]

**Table 4:** Effects of dialysis on major plasma electrolytes and glucose.

Group	Sodium (mmol L^−1^)	Potassium (mmol L^−1^)	Chloride (mmol L^−1^)	Calcium ion (mmol L^−1^)	Glucose (mmol L^−1^)
Before dialysis	139 (138–140)	3.8 (3.8–3.9)	103 (103–103)	1.28 (1.27–1.34)	12.6 (10.4–14.5)
60 min	138 (138–139)	4.2 (4.1–4.3)	103 (102–104)	1.32 (1.31–1.34)	10.2 (9.8–12.2)
120 min	138 (137–138)	4.7 (4.5–4.7)	103 (101–104)	1.33 (1.30–1.36)	7.8 (5.6–9.4)
180 min	138 (137–138)	4.5 (4.3–4.7)	103 (101–104)	1.27 (1.25–1.30)	8.2 (6.9–9.1)
30 min after dialysis	138 (137–138)	4.2 (4.0–4.7)	102 (101–103)	1.22 (1.21–1.26)	9.6 (8.2–9.9)
Asymptotic Friedman test					
P-value	0.23	0.01	0.11	0.004	0.0002
95% CI for dialysis effect	[− 0.3 to 0.4]	[0.2 to 0.7]	[− 0.1 to 0.5]	[0.37 to 0.75]	[− 0.9 to − 1.0]

### Effects on acid–base and blood chemistry during dialysis

Plasma base excess increased during dialysis (Fig. 2D). In line with this, arterial pH increased from 7.42 (7.42–7.44) to 7.51 (7.49–7.52) after 180 min dialysis (P = 0.005). Arterial *p*CO_2_ was not significantly different after 180 min hemodialysis, being 4.0 (4.0–4.3) kPa before, and 4.0 (3.7–4.2) kPa after dialysis, respectively (P = 0.81). Blood hemoglobin levels were not significantly different before *versus* after dialysis, being 113 g/L (106–118) and 116 g/L (111–118), respectively (P = 1.00). Plasma potassium increased from 3.8 mmol/L (3.8–3.9) before dialysis to 4.5 mmol/L after 180 min (P = 0.03), whereas there was no significant difference in plasma sodium (Table 4).

### In-silico modeling

**Figure 3:**
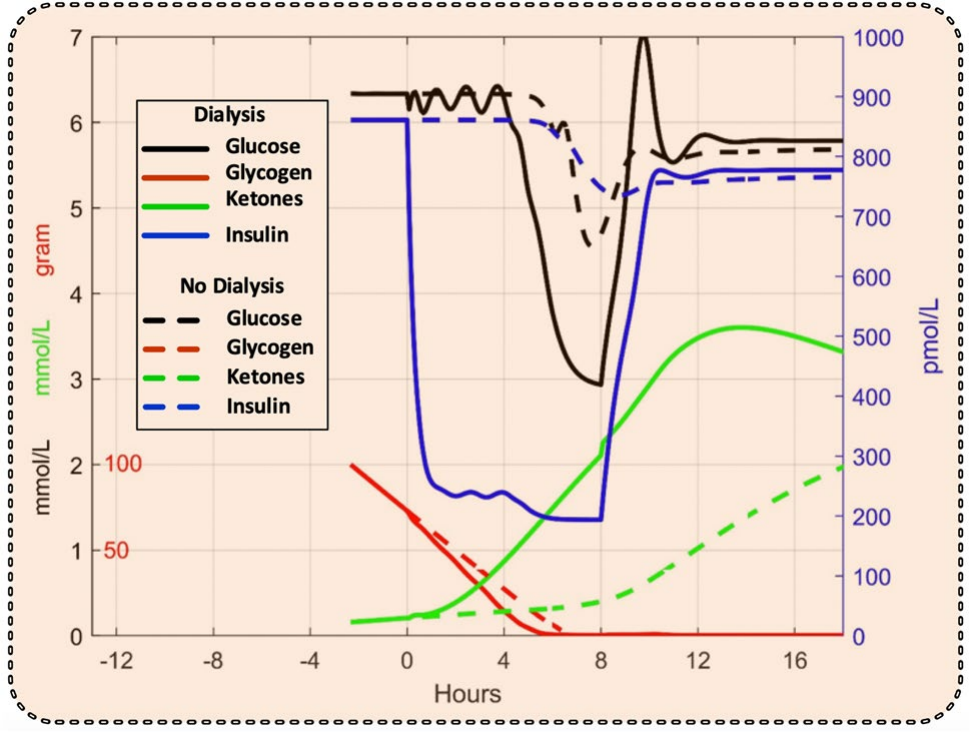
Simulated glycogen, glucose, insulin, and ketone levels without dialysis (broken lines) and before, during, and after an 8-h dialysis session started before glycogen stores have been depleted (continuous lines). Scale numbers for glucose and ketones are both in black.

**Figure 4:**
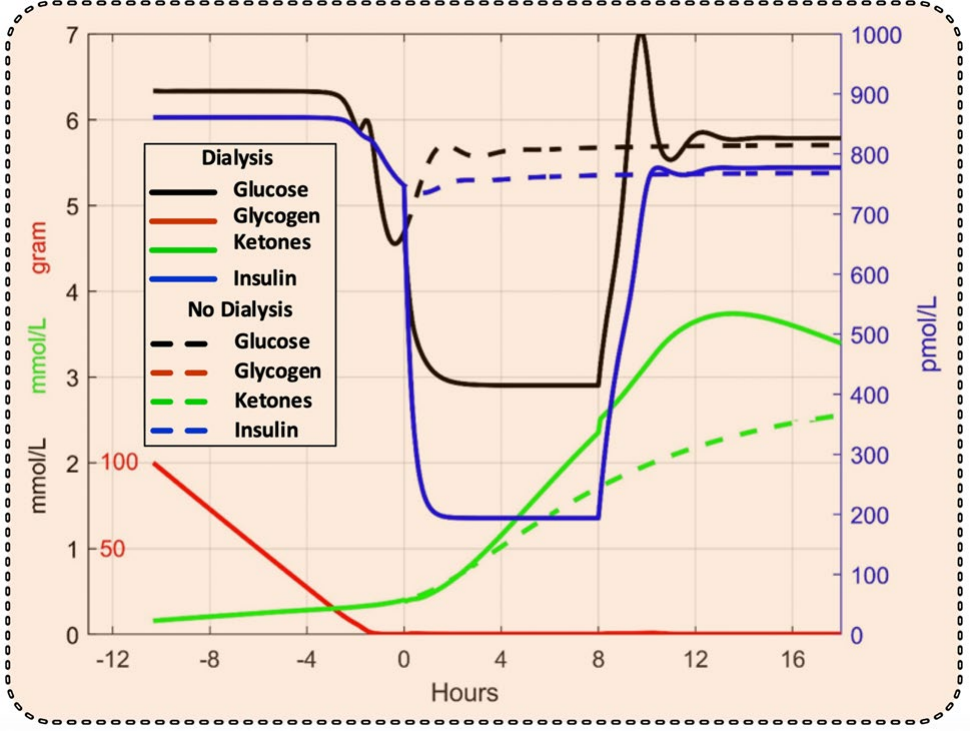
Simulated glycogen, glucose, insulin, and ketone levels without dialysis (broken lines) and before, during, and after an 8-h dialysis session started after glycogen stores have been depleted (continuous lines). Scale numbers for glucose and ketones are both in black.

The simulation in Fig. 3 showed that a significant lowering of the blood glucose level by dialysis can only be achieved after the glycogen stores in the liver have been depleted, which occurred about 3 h after the start of dialysis at time 0. Dialysis was continued for 8 h. We therefore suggest delaying the start of dialysis until this occurs, as seen in Fig. 4, where the effect of dialysis (continuous lines) was compared to the case of continued starvation without dialysis (broken lines). Dialysis reduced blood glucose and insulin levels, while the ketone levels were slightly increased.

**Figure 5:**
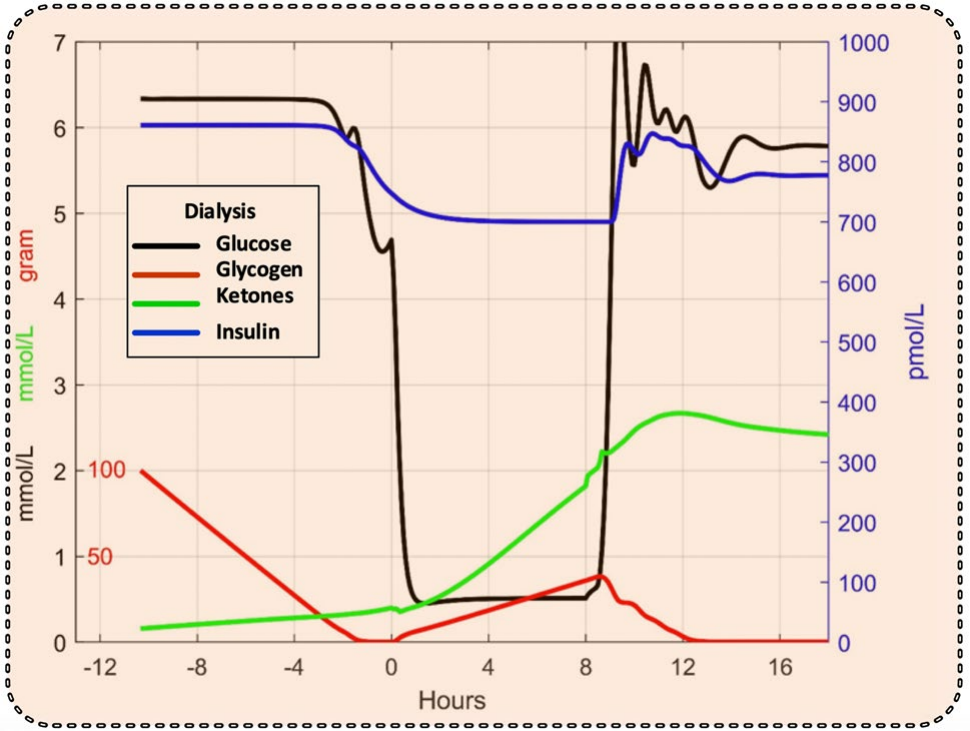
Simulated glycogen, glucose, insulin, and ketone levels before, during and after an 8-h dialysis with zero insulin clearance. Scale numbers for glucose and ketones are both in black.

In order to understand why ketone levels were increased despite the removal by dialysis we performed a second simulation where insulin clearance was set to zero. This could be approximated in the clinic by adding a suitable amount of insulin to the dialysis fluid. The results are shown in Fig. 5. Without removal by dialysis the insulin level was raised significantly, which resulted in a dramatic decrease of the glucose level. The ketone levels were slightly decreased, but were higher than without dialysis.

**Figure 6:**
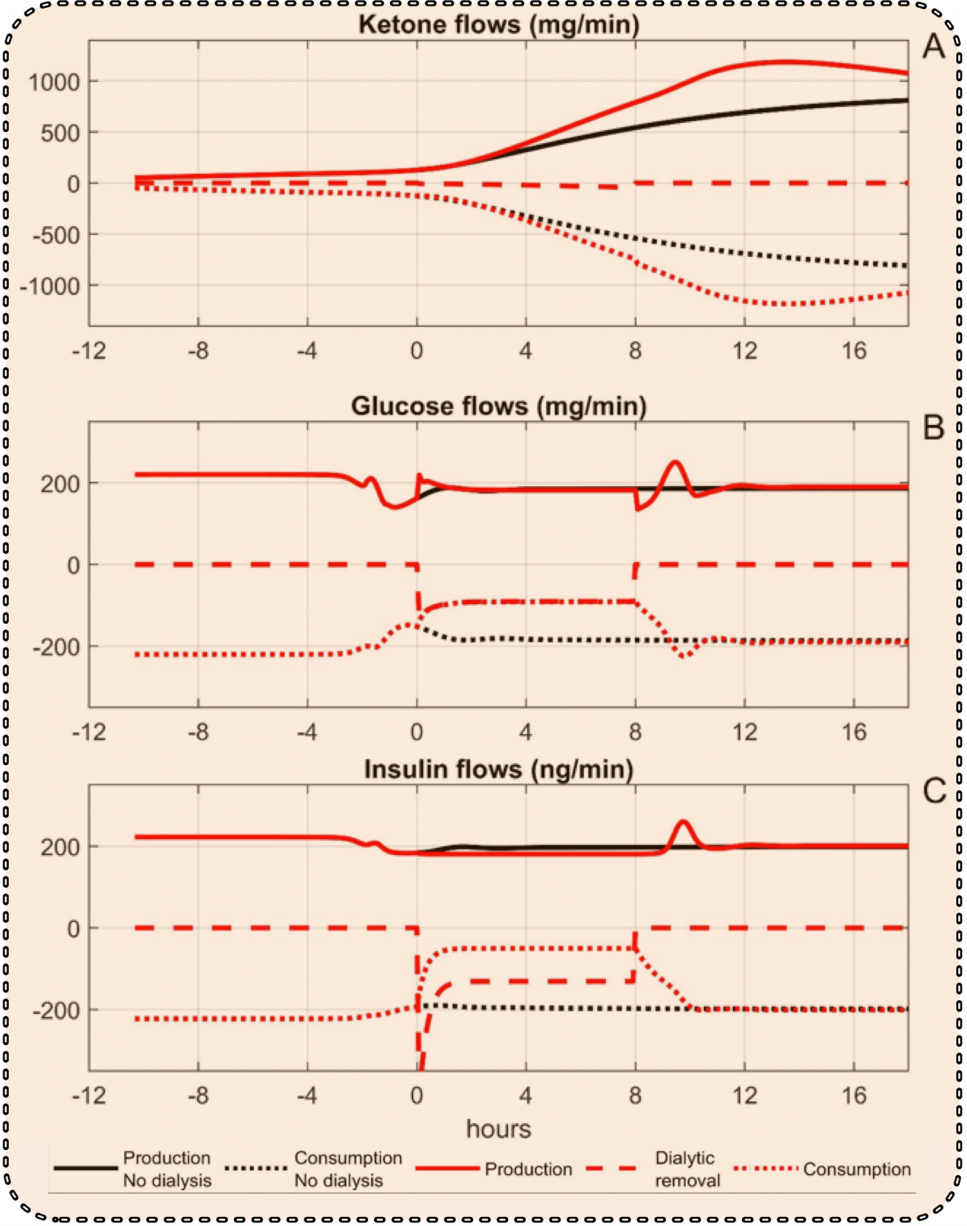
Flow rates of ketones (**A**), glucose (**B**), and insulin (**C**) to and from blood and removed by dialysis for the cases of continued starvation without dialysis (black) and with dialysis (red).

Further insight was gained by studying the transport patterns of the key substances involved. Figure 6 A shows the delivery of ketones to blood (continuous lines), the removal by dialysis (broken line), and the disappearance rate from the blood (dotted lines) for the cases of no dialysis (black), and normal dialysis (red). The removal by dialysis constituted only a small fraction of the other effects, hardly visible in Fig. 6A. Also, delivery to and removal from the blood balanced each other to a large extent, being much larger than the resulting change in the blood concentration. For glucose and insulin, shown in Fig. 6B,C, the delivery to blood was nearly unaffected by dialysis, but the removal by dialysis constituted a large fraction of the delivery to blood and the consumption was considerably reduced.

**Figure 7:**
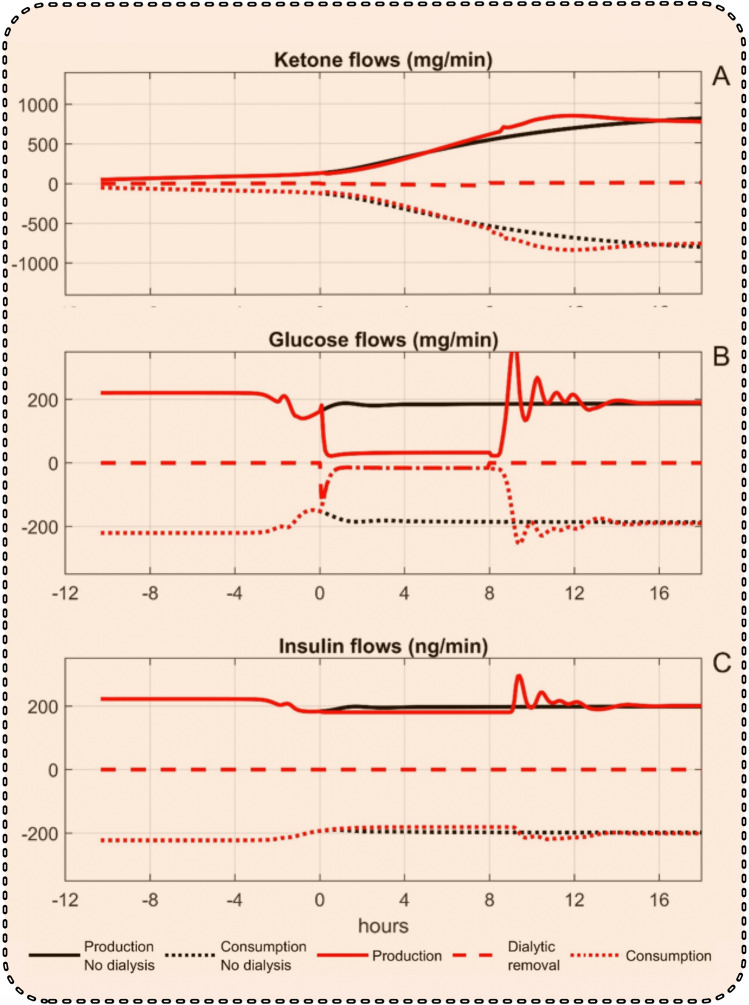
Flow rates of ketones (**A**), glucose (**B**), and insulin (**C**) to and from blood and removed by dialysis for the cases of continued starvation without dialysis (black) and dialysis with zero insulin clearance (red).

When insulin clearance was set to zero, Fig. 7A shows that the delivery and consumption of ketones were both slightly reduced, but were still larger than without dialysis. During these conditions, the turnover of glucose in Fig. 7B was very low, whereas the turnover of insulin in Fig. 7C was close to, but slightly lower than for the case with no dialysis.

## Discussion

The present study shows that blood ketone levels in rats fed with ketogenic diet were relatively unaffected by hemodialysis, despite the fact that the low molecular weight of ketones (β-hydroxybutyrate, acetoacetate, and acetone) means that they will be removed efficiently by dialysis. Indeed, molecules of the same approximate size as keto-acids were clearly effectively removed (e.g., urea), and even bigger molecules like ^51^Cr-EDTA were effectively cleared from blood. Furthermore, using a theoretical model we show that the removal of blood ketones by dialysis is most likely very small compared to the production by the liver which needs to satisfy the energy need of the entire body.

The dialysate blood flow rate (Qb) was here set to 1 mL/min, which is similar to other experimental models of hemodialysis in rats^13^. Pre-filter arterial pressures were stable and positive at this blood flow rate, with 40–50 mmHg being a typical value. In order to elucidate how this blood flow rate would correspond to a human undergoing hemodialysis it may be set in relation to the distribution volume. For example, for urea the distribution volume is approximately equal to total body water (TBW), being about 70% of body weight in rats, which is roughly three times the extracellular volume^14^. Thus, a 300-g rat has a TBW of 210 mL, meaning that a blood flow rate of 1 mL/min clears ~ 0.47% of the TBW from urea. For a 70-kg adult having a TBW of 42 L this corresponds to a blood flow of 42 × 0.47% = 200 mL/min. Nowadays, blood flow rates are usually 250 mL/min or higher, especially in patients treated with hemodiafiltration. On the other hand, we used a dialysate flow rate of 5 mL/min which are far higher than Qb meaning that solute transport will most likely be limited by the blood flow rather than the dialyzer membrane or dialysate flow. Indeed, in line with this, the estimated clearance of urea and the dialyzer plasma to dialysate clearance of ^51^Cr-EDTA (a much larger molecule than urea) was nearly identical, being 0.67 mL/min for urea and 0.66 mL/min for ^51^Cr-EDTA. It is reasonable to assume a very similar dialyzer clearance for glucose and ketones.

We also found that ketogenic diet leads to mild metabolic acidosis in rats; also, there was a very mild increase in plasma lactate at baseline before dialysis. A slightly elevated lactate level has previously been observed in cows fed with ketogenic diet^15^. Also, potassium levels were increased during dialysis to 4.5 mmol/L. This may possibly be due to the fact that potassium 20 mmol was added to a 5-L bag of fresh dialysate manually, which due to tolerances in volume and composition of the potassium solution may cause the actual dialysate concentration to differ from 4 mmol/L. Another possible explanation may be the lower insulin levels caused by dialysis, leading to a shift of potassium from the intra-cellular space to the circulation^16,17^. Lastly, we observed a slight hyperglycemia before dialysis which is in line with previous studies in mice^18^ in which they also noted lower insulin levels following ketogenic diet^18^.

The experimental results were corroborated by the simulations shown in Fig. 4, where ketone levels are even higher with dialysis than without. One explanation could be that the lower insulin levels caused by dialysis trigger an increased production of ketones that outweighs the removal by dialysis. This hypothesis was tested by a separate simulation where the insulin clearance was set to zero (Fig. 5). During such circumstances, the insulin level was almost as high as without dialysis, and the glucose level was dramatically reduced. The ketone level was slightly decreased (but still higher than without dialysis), probably due to the higher insulin level. Unexpectedly, the model also predicted an increase in glycogen.

The simulation model also allows the calculation of the flow rates of ketones, glucose, and insulin to and from the blood compartment and their removal by dialysis, which are shown in Fig. 6A–C. For ketones, the removal by dialysis is smaller than the transports to and from the blood which almost balance each other. Therefore, relatively small changes in production and/or utilization of ketones will outweigh any removal by dialysis. This explains why ketone levels can increase despite dialytic removal. On the other hand, for insulin and glucose the production is almost unaffected by dialysis, but the removal by dialysis is a significant fraction of the inflow to blood—and their concentrations are therefore to a large extent affected by dialysis. Setting insulin clearance to zero had a small effect on the flows of ketones (Fig. 7A), whereas both inflow and outflow of glucose (Fig. 7B) were dramatically reduced. The inflow of insulin (Fig. 7C) was almost unchanged, but the outflow was roughly restored to the non-dialysis value.

It should be noted that the simulated flow rates to blood of ketones and glucose are higher than previously reported production capacities of the liver, which are about 120 mg/min for ketones and 81 mg/min for glucose^19^. Another study by Balasse resulted in ketone production of 91 mg/min after 15–20 h fasting that increased to a steady level of 197 mg/min after 3 days^20^. The high simulated values could be a result of using a rodent model with human flow rates and volumes, since normal values for insulin are known to be higher for rodents than for humans^21^. We still believe that the simulations indicate the qualitative behavior and effects of dialysis using the proposed solutions.

This study has important limitations. First the small number of animals mean that only very large effects can be detected due to higher type 2 error rates. Also, the total daily caloric intake was not measured and the animals may have differed in how much they eat, causing variation in the data. However, all animals gained weight which means that the presence of starvation ketoacidosis is unlikely. Furthermore, as discussed above, the theoretical model used was not completely adapted to human conditions^8^.

We conclude that blood ketones were apparently unaffected by hemodialysis in rats, probably due to the fact that ketone production was far higher than dialytic clearance. The true rates of ketogenesis, gluconeogenesis, and the secretion of insulin, glucagon, and other hormones should be assessed in future investigations. The clinical utility of cancer dialysis should be confirmed in a clinical study.

## Data Availability

Original experimental data reported in this article have been deposited in figshare (https://doi.org/10.6084/m9.figshare.21769886).
